# Miscellaneous

**Published:** 1854-11

**Authors:** 


Congestion of the brain in cholera, by James M. Newman, M. D., Buffalo, N. Y.

Galt on Insanity.

Elkoplasty or Anaplasty applied to the treatment of old ulcers; also a new mode of treatment for delayed or non-union of a fractured humerus, by Frank H. Hamilton, A. M., M. D., Professor of Surgery, &c., &c.

Resume de Recherches cliniques sur la fievre continue, la dyssenterie, la pleuresie chronique, et sur les variations du ton dans les sons fournis par la percussion, et par l'auscultation, par Austin Flint, M. D., Professeur de Medicine, theorique et pratique a l'Universite de Louisville, etat de Kentucky, Etat Unis d'Amerique. Paris. 1854.

Proceedings of the American Pharmaceutical Association, at the third annual meeting held in Cincinnati, July 25th and 26th, 1854. Published by direction of the Association.

Eighth Annual Report of the Board of Regents of the Smithsonian Institution.

Dr. Newman enumerates the following as the premonitory and positive symptoms of congestion of the brain in cholera:

There is a class of cases in which there never occurs the pallid, sunken countenance generally seen in cholera, and so characteristic of the disease. If reaction is established, the bright, if not ruddy complexion of the patient seems to belie the assertion that but a few hours before they were at the very mouth of the grave.

This redness of the face is attended by several peculiarities. In some cases it presents the appearance of a bright blush diffusing itself over the whole cheek. In others it is a deeper red, and not so largely diffused; the color toward the center of the patch becoming more and more intense, a bright circumscribed spot is manifest upon a field of less intensity. The small cutaneous vessels are minutely injected, and the general appearance is not unlike that caused by the immoderate use of alcoholic stimulants, or frequent and long exposure to the sun.

This redness of the skin, depending upon the injection of the vessels, is very persistent. It remains during the whole period of treatment, and long after convalescence. It seems as if it required to be removed by some process of reparation.

The eyes are more or less injected. The intensity of this injection is very various, and is increased with the the persistency of the case, and its tendency to a fatal termination. The conjunctivæ in some cases are injected in their lower halves only, that portion covered by the upper lids remaining singularly clear.

If convalescence be not speedily established, the vomiting and purging continue, or, if for a time checked return again. Drinks, medicines, and nourishments are all alike rejected by the stomach; and the matters thrown up cease to be a mere colorless liquid, but are more or less tinged, generally being yellow, sometimes greenish.

The discharges from the bowels are frequent and assume in a greater or less degree the consistency, odor and appearance of ordinary diarrhœa. The discharges sometimes are very offensive. This condition is not always observable, the evacuations occasionally continuing throughout the disease rice-water. But I believe this is more generally observable in cases of a rapid termination, and in cases where a relapse has been sudden from a condition of comparative convalescence, and partakes of the character of a second attack of cholera.

As the vomiting and diarrhœa continue, the strength wanes; the color of the face deepens, or extends in surface; the eyes are more and more injected: the patient becomes drowsy and inclined to sleep; the stupor gradually increases, and finally profound coma becomes established, terminating in death, or recovery after long treatment and a tedious convalescence.

The condition just described is Conjestion of the Brain in the course of Cholera.

The flushed face, and injected eye, are the premonitory symptoms of and the indications pointing out the tendency of the disease to such a termination, and warn us against any treatment which may precipitately hurry it on, and conduce to hasten our patients to the grave.

I would solicit for the consideration of these conditions of the face and eye, more than a casual notice. I have come to regard them as valuable diagnostic signs; and as they occur earlier in the disease, look upon them as affording us an unerring index to our course of treatment. With these marks upon our patients we have Congestion of the Brain, in some form or other, to contend with. And fortunate indeed shall we be if our patient prove to be lightly affected, or our medication wards off the impending evil.

The author regards the vomiting and purging at this stage of the disease as choleraic only in their character and indications, the vomiting depending on irritation of the brain from congestion, while the diarrhœa is due partly to the same cause, and partly to the exudation of the serum of the blood through the coats of the intestines, in consequence of a change of vital action or lesion of structure in their membranes. Although the writer regards this as a stage of cholera, yet he says that in the treatment "we are here to cease to regard and to treat the disease as cholera merely but we are to merge this consideration into a knowledge of the fact that we have a brain affection to treat sufficiently serious to destroy our patient." Grant this fact, but what is this brain affection? Dr Newman thinks it may be simple conjestion or a state of inflammation co-existing with congestion. It has been supposed by others that it is dependent on retained excretible materials, especially urea, acting as a positive poison to the nervous system. It has seemed to us from many observations that it is simply a local manifestation of the *grand mal*, and that to understand it we have only to trace the changes that take place in the fluids and solids of the body from the first invasion of the disease.

The premonitory diarrhœa, as it has been called, we consider one stage of cholera. During this period the water of the blood is rapidly passing into the alimentary tube; the specific gravity of the liquor sanguinis in the blood vessels is increased; and by the law of exosmosis, the contents of the corpuscles passes through their walls, leaving them in a corrugated condition. As the disease progresses, first the inorganic, second the organic solids pass into the intestinal canal, the blood corpuscles become still more collapsed, from the viscid condition of the blood, they circulate but slowly, and their function as the carriers of oxygen is performed with constantly increasing difficulty.

We believe it is admitted by physiologists of the present day that the functional activity of parts is dependent on the molecular changes in their ultimate organic elements, and that these changes are effected mainly by the agency of oxygen. If this be so, we can understand how a series of morbid changes in the fluids and solids of the blood may affect the different extra-vascular structures. The old worn-out particle is not removed, the new material is not supplied, the process of secondary assimilation is entirely arrested, with a consequent loss of function, not of one organ only but of the whole system. The arrest of the circulation or the congestion seems therefore to be dependent on two causes: first, the viscidity of the blood, from a loss of water; secondly and chiefly, in the loss of the force existing in the capillaries and in the arterial walls. It is general in its character, manifesting itself, first through those organs most sensitive to injury, and in their derangements affecting most seriously the vitality of the organism. The therapeutical indications are to restore to the blood its normal conditions, and to excite the secretions by introducing into the system those substances known to possess an influence over them.

The first of these indications may be fulfilled by allowing or requiring, if need be, that the patient drink largely of weak solutions of the neutral salts of soda and potassa, while at the same time beef tea or the essence of beef is administered in as liberal quantities as the stomach will retain, thus supplying those constituents that have been drained during the active stage of the disease. For meeting the second indication, we may use in addition to the salines above mentioned, mercurials, the measure of their quantity being the effect produced. The experience of the profession we believe, has furnished quite unanimous testimony to their good effects in this stage of the disease, but whether they act as revulsives as Dr. Newman thinks, and thus relieve the congestion which he seems to suppose is confined to the brain, or whether they serve as excitants of the organic actions generally, as we think more probable, it is perhaps difficult to say.

The essay of Dr. Galt is from the American Journal of Insanity. It contains much that is interesting, not only to those who are more immediately charged with the care of the Insane, but also to the general medical reader.

Dr. Hamilton proposes to cure indolent ulcers, where there is a loss of integuments, by grafting from remote parts healthy tissues. The following is his summary:

1st.—Ulcers, accompanied with extensive loss of integument, do generally refuse to heal, whatever may be the health of the body or of the limb.

2d.—Anaplasty will sometimes succeed in accomplishing a permanent cure, and especially where the health of the body and of the limb are perfect, and where, by inference, the refusal to heal is alone attributable to the extent of the integumentary loss.

3d.—The graft must be brought from a part quite remote; generally from an opposite limb, or from another person.

4th.—If smaller than the chasm which it is intended to fill, the graft will grow, or project from itself new skin to supply the deficiency.

5th.—It is not improbable that the graft will expand during the process of cicatrization at its margins, but especially for a time after the cicatrization is consummated.

6th.—In consequence of one or both of these two latter circumstances, it will not be necessary to make the graft so large as the deficiency it is intended to supply.

Dr. H. proposes to treat ununited fractures of the humerus by first straightening the arm. A case is reported in which this course was pursued and the ends of the bones perforated, as recommended by Dr Brainard in similar cases. Union was established in less than forty days.

Dr. Flint, in his resumé, has given the essential points of his work on continued fever, dysentery, chronic pleurisy, and his prize essay on the variations of the pitch in the sounds furnished by auscultation and percussion, published in French during his recent visit to Paris. The works have been noticed in our journal, and we have nothing more to say in reference to them.

We have not space in the present number to do more than acknowledge the receipt of the remaining works.

We have received catalogues of medical and surgical works, by Blanchard & Lea, Philadelphia; and the Messrs. Wood of New York. Also, the Eighteenth Annual Report of the Directors and Superintendent of the Vermont Asylum for the Insane. J.

